# Reliability and Concurrent Validity of a Chinese Version of the Alberta Infant Motor Scale Administered to High-Risk Infants in China

**DOI:** 10.1155/2018/2197163

**Published:** 2018-06-13

**Authors:** Hui Wang, Haifeng Li, Jiangping Wang, Huiying Jin

**Affiliations:** The Pediatric Rehabilitation Department, Children's Hospital, Zhejiang University School of Medicine, Hangzhou, Zhejiang, China

## Abstract

The Alberta Infant Motor Scale (AIMS) is widely used to screen for delays in motor development in high-risk infants, but its reliability and validity in Chinese infants have not been investigated. To examine the reliability and concurrent validity of AIMS in high-risk infants aged 0-9 months in China, this single-center study enrolled 50 high-risk infants aged 0-9 months (range, 0.17-9.27; average, 4.14±2.02), who were divided into two groups: 0-3 months (n=23) and 4-9 months (n=27). A physical therapist evaluated the infants with AIMS, with each evaluation video-recorded. To examine interrater reliability, two other evaluators calculated AIMS scores by observing the videos. To measure intrarater reliability, the two evaluators rescored AIMS after >1 month, using the videos. Concurrent validity was assessed by comparing results between AIMS and the Peabody Developmental Motor Scale-2 (PDMS-2). For all age groups analyzed (0-3, 4-9, and 0-9 months), intraclass correlation coefficients (ICCs) for AIMS total score were high for both intrarater comparisons (0.811-0.995) and interrater comparisons (0.982-0.997). AIMS total scores were well correlated with all PDMS-2 subtest scores (ICC=0.751-0.977 for reflexes, stationary, locomotion, grasping, and visual-motor integration subsets). However, the fifth percentile of AIMS total score was only moderately correlated with the gross motor quotient, fine motor quotient, and total motor quotient subtests of PDMS-2 (kappa=0.580, 0.601, and 0.724, respectively). AIMS has acceptable reliability and concurrent validity for screening of motor developmental delay in high-risk infants in China.

## 1. Introduction

Certain infants are considered to be at high risk of growth and developmental delay during the prenatal, intrapartum, and postnatal periods. These high-risk infants are particularly vulnerable to cerebral injury and abnormal brain development [1, 2], which can result in permanent sequelae such as cerebral palsy and intellectual disability. Neuronal plasticity is enhanced in the developing brain, particularly during the first year of an infant's life [3]. The early detection of motor deficits and developmental delay in an infant allows for appropriate interventions to be instigated at the earliest possible opportunity, maximizing the potential for clinical benefit. Indeed, early intervention with suitable programs has been reported to have a positive effect on motor development in infants with or at high risk of developmental delay [4]. Clinical assessments of high-risk infants provide important guidance and support to the caregivers during the critical first year of an infant's life. However, it is crucial that these assessments are able to identify infants with developmental delay as accurately and as early as possible.

Peabody Developmental Motor Scales-2 (PDMS-2) is an alternative assessment for children aged 0–6 years [5] that is standardized and normed for infants/toddlers/children in the USA. PDMS-2 is a comprehensive motor function assessment scale widely used in high-risk infants. Previous studies have provided evidence for the reliability and concurrent validity of PDMS-2 when used to assess motor development and identify motor deficits in high-risk infants [6–8]. PDMS-2 has also been used to assess high-risk infants under Chinese socioeconomic and cultural constraints [9]. Since PDMS-2 has been shown to be a reliable and validated tool [5, 10–13], it is widely used in China as a discriminative measure for evaluating motor development in the clinical setting. However, PDMS-2 contains a large number of assessment items and requires a long administration time of 45–60 minutes. This is a major disadvantage in China, where the population of high-risk infants has gradually increased [14] and the demand for assessment is high. Indeed, the limited medical and social resources in China mean that it would be practically challenging to use PDMS-2 to screen all infants at high risk of motor development delay. Thus, alternative screening tools are needed that are not only simple and quick to administer but also reliable and validated in the clinical setting.

The Alberta Infant Motor Scale (AIMS) is an assessment tool that measures the motor maturation of infants from birth to the age of independent walking and incorporates the neuromaturational concept and dynamic systems theory [15]. AIMS was originally developed to screen motor development in Canadian full-term and preterm infants [15]. Although not as comprehensive as PDMS-2, major advantages of AIMS are that it is straightforward to administer, its use involves only observation, and the assessment can be completed within 20 minutes. Because of its practicality and psychometric characteristics, AIMS is widely used in the clinical setting [16]. The reliability and validity of AIMS have been investigated in infants in Canada, Brazil, Japan, and Taiwan [16–20]. However, no previous studies have examined the reliability and concurrent validity of AIMS when used to screen high-risk infants in China.

The purpose of this study was to investigate the intrarater and interrater reliabilities and concurrent validity (compared to PDMS-2) of AIMS when used by physical therapists to screen high-risk infants in China.

## 2. Study Design and Participants

### 2.1. Study Design

This was a prospective study carried out at the Pediatric Rehabilitation Outpatient Department of the Children's Hospital Affiliated to Zhejiang University School of Medicine, Hangzhou, Zhejiang, China, between October 2013 and December 2013. A total of 50 infants aged 0–9 months, who were at high-risk of developmental delay, were enrolled in the study based on the inclusion and exclusion criteria. The inclusion criteria were (i) age (corrected for gestation) ≤9 months and (ii) being at high risk of developmental delay due to the presence of one or more of the following factors: low birth weight (<2500 g), prematurity (gestational age <37 weeks), polyembryony, intrauterine infection, intrauterine hypoxia, hypoxic-ischemic encephalopathy, asphyxia, neonatal hyperbilirubinemia, neonatal intracranial hemorrhage, or use of a ventilator due to lung dysplasia. The exclusion criteria were (i) visual or auditory impairment, (ii) hereditary metabolic diseases or myogenic diseases, and (iii) congenital malformations or severe congenital heart disease.

The study was approved by the Ethics and Human Research Committees of the Children's Hospital of Zhejiang University School of Medicine. Written informed consent was obtained from the parents/guardians of all infants before their inclusion in the study.

### 2.2. Participants

The total sample consisted of 21 girls and 29 boys, corrected age from 5 days to 9 months. For assessments of the reliability of AIMS, the study participants were divided into two groups based on age (0–3 months, n = 23; 4–9 months, n = 27) to ensure a relatively equal representation of different levels of motor performance. For the purposes of this study, infant age was taken as the age corrected for gestation.

The following demographic and clinical characteristics were recorded: gender, age, gestational age, birth weight, length of hospital stay, and the presence/absence of polyembryony, birth asphyxia, intracranial hemorrhage, hyperbilirubinemia, and use of a ventilator.

## 3. Selection of Raters

The three raters (A, B, and C) were rehabilitation therapists with more than 3 years of professional experience, including extensive experience of pediatric patients and administration of development assessment scales. All raters had received specialized training at the Rehabilitation Department in infant motor development theories and the use, application, and scoring of AIMS. During training, the raters were provided with instructions and demonstrations of the AIMS testing procedures and rating criteria. Following the training session, the raters were required to administer the AIMS to 10 high-risk infants (the data obtained from the infants examined during the training sessions were not included in the final analyses of reliability and validity); the consistency of the AIMS scores exceeded 0.8, which was deemed satisfactory.

The PDMS-2 assessment was carried out by a chief physician in child rehabilitation, who had 8 years of work experience, extensive knowledge of child development theory and child assessment theory, and substantial practical experience in the administration of PDMS-2.

## 4. Process of Infants' Evaluation with Both Tools

### 4.1. Overall Procedure

All infants were initially given AIMS on-site by rater A, while a videographer video-recorded the infant's performance throughout the examination. Following this, on the same day, a rehabilitation physician completed the PDMS-2 assessment [5], which was used to investigate concurrent validity. These evaluations were carried out in a quiet, undisturbed, well-lit room at a temperature of 20–30°C; the children were clothed in one or two layers and encouraged to play at their best level.

Subsequently, raters B and C (who were blinded to the evaluation of rater A) independently evaluated the AIMS score for each infant by observation of the video recordings; these scores and the scores by rater A were used in the assessment of interrater reliability. Raters B and C reevaluated the AIMS score for each infant one month later (again by observation of the video recordings); these scores were used in the assessment of intrarater reliability. A time interval of one month was considered long enough to minimize the memory bias of the rater. Due to the use of a video-recorded evaluation, raters B and C did not have to handle the child, which eliminated one potential source of error.

### 4.2. AIMS

AIMS is a behavioral motor assessment tool that requires careful observational techniques and minimal infant handling. The scale consists of 58 items that are categorized by four subscales: prone (21 items), supine (9 items), sitting (12 items), and standing (16 items) [15]. For each test item, the examiner must identify and observe three key descriptors: weight bearing, posture, and antigravity movement. The sum of the observed criteria for each subscale comprises the total raw score (0–58 points). The final raw scores can be converted into percentile ranks and compared with the ranks of age-matched peers. Infants below the fifth percentile are identified as having movement dysplasia [17, 21]. The Chinese version of AIMS used in the present study had been translated from the English version but had not been subjected to cultural adaptation.

## 5. PDMS-2

We used PDMS-2 as a standard against which to examine the concurrent validity of AIMS. PDMS-2 consists of a gross motor scale and fine motor scale, each of which is divided into skill subtests that detect typical motor tasks for each age. Test items are scored on a scale of 0–2 points with a score of 1 indicating partial success. The performance of the test piece is summarized and analyzed by employing motor quotients derived by adding the subtest standard scores and converting the sum to a quotient that has a mean value of 100 and a standard deviation of 15. The motor quotients include the gross motor quotient (GMQ), fine motor quotient (FMQ), and total motor quotient (TMQ: comprised both GMQ and FMQ). The GMQ includes reflexes (RE, for infants aged 0–12 months) or object manipulation (OB, for infants aged >12 months), and stationary (ST) and locomotion (LO) subtests, while FMQ includes grasping (GR) and visual-motor integration (VI) subtests. Motor quotient scores <90 were interpreted as indicative of movement dysplasia.

## 6. Data Analysis

Statistical analysis was performed using SPSS16.0 (SPSS Inc., Chicago, IL, USA) and MedCalc Software (MedCalc 9.2.10, Belgium) for Bland-Altman analysis. Data were tested for normality. Normally distributed data are presented as means ± standard deviations (SDs), nonnormally distributed data as medians and ranges or interquartile ranges (IQRs), and categorical data as n (%). Intrarater and interrater reliability were examined by calculation of the intraclass correlation coefficient (ICC) and the 95% confidence interval (95%CI) of the ICC. Interrater ICCs were calculated on the subsections and the total scorings of AIMS for each age group by three raters (A, B, and C) when assessing the same infant; the ICC value between each pair of raters was also calculated. Intrarater ICCs were calculated on the repeated scorings in one-month interval by rater B and rater C. Interrater and intrarater reliability were also analyzed using Bland-Altman plots. Because of the high degree of correlation between the development of gross motor and fine motor skills [22], concurrent validity was assessed also by calculation of the ICC and the 95%CI of the ICC between the AIMS total raw score and the PDMS-2 raw score for each subtest (including gross motor and fine motor). The kappa concordance coefficient was calculated to analyze the qualitative consistency between the AIMS percentile and the PDMS-2 GMQ, FMQ, and TMQ scores. As described by Portney and Watkins [23], ICCs of >0.90, 0.75–0.90, 0.50–0.75, and <0.5 were taken to indicate high, good, moderate, and poor reliability, respectively. Kappa values of >0.75, 0.40–0.75, and <0.40 were taken to indicate good, moderate, and poor consistency, respectively.

## 7. Results

### 7.1. Demographic and Clinical Characteristics of the Study Participants

A total of 50 infants (21 females and 29 males) with an average age of 4.14 ± 2.02 months (range, 0.17–9.27 months) were included in the study. The demographic and clinical characteristics of the study participants are listed in**Table 1**.

### 7.2. Interrater Reliability

The AIMS total and subsection (prone, supine, sitting, and standing) scores recorded independently by each rater (A, B, and C) and analyzed for all patients as well as for subgroups based on corrected age (0–3 months and 4–9 months) are shown in**Table 2**. The interrater ICC values are also listed in**Table 2**. The interrater ICC value exceeded 0.9 across all age groups for all AIMS subsections (ICC range: 0.920–0.997). The interrater ICC value for total AIMS score was 0.982 (95%CI: 0.963–0.992) for patients aged 0–3 months, 0.996 (95%CI: 0.992–0.998) for patients aged 4–9 months, and 0.997 (95%CI: 0.995–0.998) for all patients. The reliability of AIMS was further examined between each pair of raters (**Table 3**). The ICC value between each pair of raters exceeded 0.9 across all age groups for all AIMS subsections, except for the standing subsection scores (across all age subgroups) for rater C versus rater A or B. The overall ICC (total AIMS score for all patients) was 0.999 (95%CI: 0.997–0.999) for rater A versus B, 0.994 (95%CI: 0.989–0.997) for rater A versus C, and 0.994 (95%CI: 0.990–0.997) for rater B versus C.

**Figure 1:**
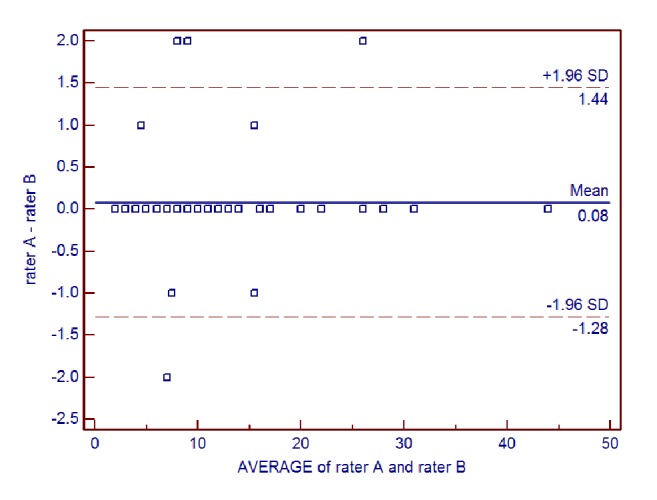
Bland-Altman plot of interrater reliability of the Alberta Infant Motor Scale (AIMS) total score for rater A and rater B.

**Figure 2:**
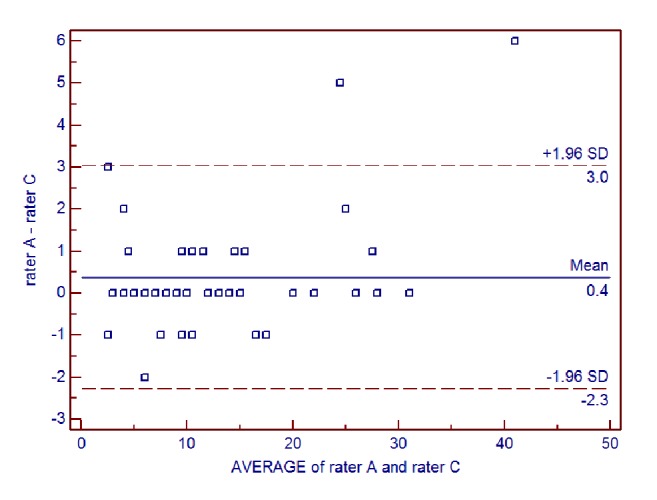
Bland-Altman plot of interrater reliability of the Alberta Infant Motor Scale (AIMS) total score for rater A and rater C.

**Figure 3:**
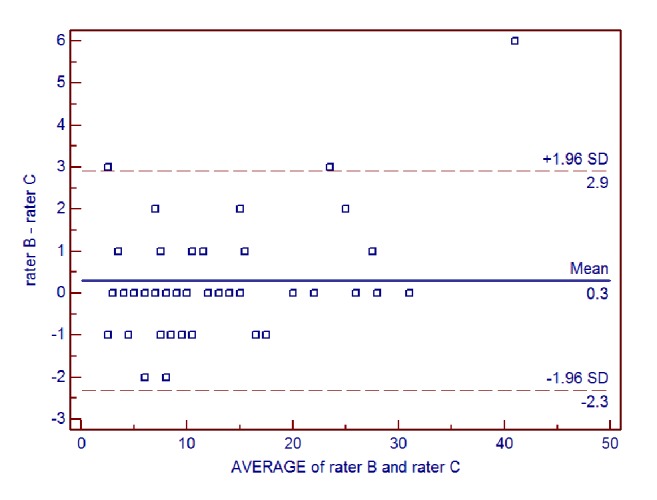
Bland-Altman plot of interrater reliability of the Alberta Infant Motor Scale (AIMS) total score for rater B and rater C.

Interrater reliability of the AIMS total score for all patients between each pair of raters was further examined using Bland-Altman analysis (**Figures 1, 2, and 3**). For rater A and rater B, the mean difference was 0.08, the SD of the differences was 0.70, and the lower and upper limits were -1.28 and 1.44, respectively. For rater A and rater C, the mean difference was 0.38, the SD of the differences was 1.35, and the lower and upper limits were -2.27 and 3.03, respectively. For rater B and rater C, the mean difference was 0.30, the SD of the differences was 1.33, and the lower and upper limits were -2.31 and 2.91, respectively.

### 7.3. Intrarater Reliability

The intrarater ICC values for AIMS total score and subsection scores (prone, supine, sitting, and standing) are shown in**Table 4** for rater B and**Table 5** for rater C. For raters B and C, respectively, intrarater ICC values for total AIMS score were 0.872 (95%CI: 0.699–0.946) and 0.811 (95%CI: 0.554–0.920) for infants aged 0–3 months, 0.991 (95%CI: 0.980–0.996) and 0.992 (95%CI: 0.983–0.997) for infants aged 4–9 months, and 0.995 (95%CI: 0.995–0.998) and 0.994 (95%CI: 0.989–0.996) for all infants. The ICC values for both raters were generally lowest for the standing subsection of AIMS in both age groups. Furthermore, the ICC values for the subsection scores were generally lower for the 0–3 months' age group (0.580–0.869) than for the 4–9 months' age group (0.728–0.991).

**Figure 4:**
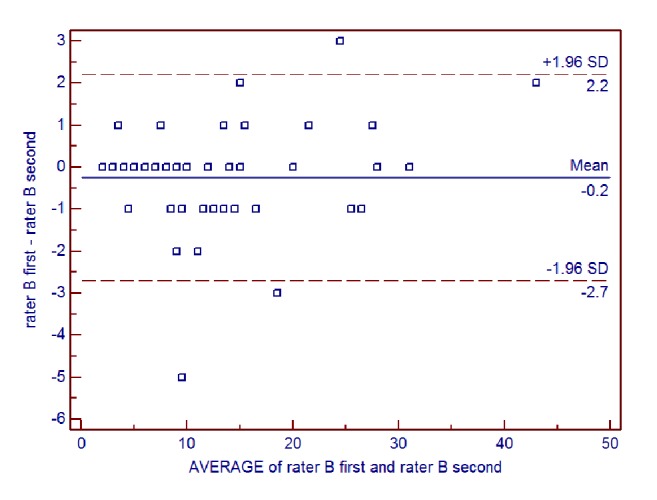
Bland-Altman plot of intrarater reliability of the Alberta Infant Motor Scale (AIMS) total score for rater B.

**Figure 5:**
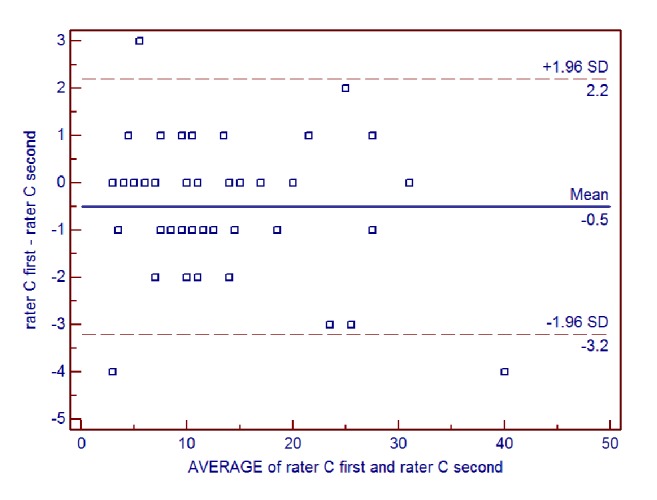
Bland-Altman plot of intrarater reliability of the Alberta Infant Motor Scale (AIMS) total score for rater C.

Intrarater reliability of the AIMS total score for all patients was further examined using Bland-Altman analysis (**Figures 4 and 5**). For rater B, the mean difference between ratings was -0.24, the SD of the differences was 1.25, and the lower and upper limits were -2.70 and 2.22, respectively. For rater C, the mean difference between ratings was -0.50, the SD of the differences was 1.37, and the lower and upper limits were -3.19 and 2.19, respectively.

### 7.4. Concurrent Validity

PDMS-2 was administered to 47 infants, for whom the AIMS total score was 13.68 ± 8.95; 3 infants were not assessed with PDMS-2 because they were <1 month of age. The PDMS-2 raw scores for these 47 infants and the ICC value between the AIMS total score and the PDMS-2 raw scores for the RE, ST, LO, GR, and VI subtests are shown in**Table 6**. The ICC value exceeded 0.9 for all PDMS-2 subtests, except for the RE subtest scores (ICC: 0.751, 95%CI: 0.553–0.861). Correlation analysis suggested a good positive correlation (all ICCs> 0.75).

The correlation coefficients between the fifth percentile of the AIMS total score and each of GMQ, FMQ, and TMQ are presented in**Table 7**. In this assessment of qualitative consistency, 17 high-risk infants were on or below the fifth percentile of the AIMS total score, while 30 infants were above the fifth percentile. GMQ was <90 in 16 infants and ≥90 in 31 infants; FMQ was <90 in 11 infants and ≥90 in 36 infants; and TMQ was <90 in 17 infants and ≥90 in 30 infants. The kappa concordance correlations of the AIMS fifth percentile with GMQ, FMQ, and TMQ were 0.580, 0.601, and 0.724, respectively, suggesting a moderate correlation.

## 8. Discussion

An important finding of our study was that the intrarater and interrater reliability of total and the various subsections of AIMS score were high in infants with a corrected age of 9 months or less. In addition, AIMS total score was well correlated with the various PDMS-2 subtest scores (ICC: 0.751–0.977), although the fifth percentile of AIMS total score was only moderately correlated with the GMQ, FMQ, and TMQ subtests of PDMS-2 (kappa values of 0.580–0.724). Overall, our study provides evidence that AIMS shows excellent reliability and concurrent validity when used to screen for motor developmental delay in infants aged ≤9 months in China.

The findings of our study regarding the reliability of AIMS were similar to those reported previously in many countries, not only for normally developing infants [15, 19, 20, 24–26] but also for high-risk infants [27]. Thus, numerous previous investigations have demonstrated high levels of intrarater and interrater reliability when AIMS is used to evaluate motor development in both normally developing infants and high-risk infants.

It was reported previously that the AIMS scores of infants at dual risk of motor delays or disabilities were very similar between novice examiners and experienced examiners in the USA (ICC values of 0.98–0.99) [28]. This suggests that it is relatively straightforward to rapidly train medical staff in the correct administration of AIMS. Our study also indicated that AIMS could be reliably and easily administered by physical therapists to high-risk infants in China after only a short training course in the theories of motor development and the use of AIMS. When each of the AIMS subtests (prone, supine, sitting, and standing) were examined independently for the different age groups (0–3 months, 4–9 months, and 0–9 months), the interrater ICC values between any two raters were all >0.92 (and many values were ≥0.98), with the exception of interrater ICC values for the standing subtest, which were notably lower (rater C versus A or B: 0.880 for ages of 0–3 months, 0.814 for ages of 4–9 months, and 0.867 for ages of 0–9 months). Intrarater reliability for the standing subtest was also low for both raters, particularly for younger infants (0.580–0.754 for ages 0–3 of months, 0.728–0.922 for ages of 4–9 months, and 0.819–0.923 for ages of 0–9 months). These findings are consistent with those reported previously in Canada [15] and China [26]. The lower reliability for the standing subtest of AIMS in younger infants, particularly those aged 0–3 months, may reflect the difficulty in assessing standing movements in young infants and thus greater variability between scores than for other subtests. Furthermore, younger infants are able to perform fewer items than older infants, which may also contribute to the weaker correlations observed in infants aged 0–3 months. Nonetheless, the overall interrater and intrarater ICC values exceeded 0.99, confirming that AIMS is a very stable and reliable method for evaluating motor development in high-risk infants.

A validity study in Canada found a high correlation between AIMS and PDMS-2 (a correlation coefficient of 0.99) when the tests were concurrently applied on full-term infants [15]. Similarly, a report in China determined that the correlation coefficient between AIMS and PDGMS-2 in high-risk infants aged 1–9 months reached 0.91 [29]. Our results also showed high degrees of correlation between the AIMS score and all the PDMS-2 subscale scores in high-risk infants. The correlation coefficients between total AIMS score and each of the PDMS-2 subtest scores (RE, ST, LO, GR, and VI) were all above 0.75, with the highest correlation being between the PDMS-2 LO subscale and AIMS total score. These findings support the concurrent validity of AIMS and PDGMS-2, particularly for the LO subscale, in agreement with a previous study in the USA [28]. Gross and fine motor skills in infancy are important parts of human intelligence and depend on the development of feeling and cognition [30]. The development of gross motor skill is assessed by the RE, ST, and LO subtests of PDMS-2. RE represents a fundamental basis of gross motor development in infants because of its complex involvement in the regulation of ST and LO by the nervous system. Fine motor skills are developed by accessing basic ST and LO abilities. Visual function also affects the development of ST and LO, which together promote the development of fine motor abilities [22]. Therefore, gross motor development is closely related to fine motor development, with each promoting the other. A high degree of correlation between AIMS and PDFMS-2 was found in our study, which suggests that AIMS is a reliable motor assessment scale for high-risk infants. However, the correlations between the fifth percentile of AIMS and the motor quotients of PDMS-2 (GMQ, FMQ, and TMQ) were only moderate-to-good. This discrepancy may be due to sampling bias. Standardization of AIMS was established in infants in Canada, and being below the fifth percentile was considered to be indicative of motor dysplasia, while PDMS-2 was designed using American norms, with the lowest 12% considered as having motor dysplasia. Furthermore, different approaches to correcting for gestational age between the two scales (40 weeks for AIMS and 37 weeks for PDMS-2) may also have contributed to the moderate degree of correlation between the fifth percentile of AIMS and the motor quotients of PDMS-2.

Screening motor development in high-risk infants in China is a challenging task because of the large population and relative lack of medical resources. Therefore, it is imperative to select an infant motor assessment scale that can monitor motor development and detect motor disorders in high-risk infants with high sensitivity. AIMS has the important advantages that it is straightforward to administer and requires observation of the infant only. Furthermore, AIMS is sensitive in identifying children with subtle movement problems and could potentially identify motor deficits in high-risk infants at an early stage. The reliability and concurrent validity of AIMS determined in our study suggest that physical therapists could choose either AIMS or PDMS-2 for evaluating the motor development of high-risk infants. AIMS might better fulfill the current need in the field of high-risk infant motor assessment because the process and quality of movement as well as the achievement of specific milestones are considered. Furthermore, the ease of administration of AIMS and the relatively short time required may make this instrument more feasible for use in follow-up clinics for infants at risk of motor delays.

## 9. Limitations of this Study

This was a single-center study, so the generalizability of our findings remains unknown. Furthermore, the small sample size limits the statistical power of the study. Since our evaluators participated in a training session and practiced so as to achieve a certain level of agreement, our results may not be representative of those that would be obtained by therapists in general practice. Due to the use of a video-recorded evaluation, raters B and C did not have to handle the infant, eliminating a potential source of error; in general practice, differences in handling skills between therapists may lead to lower reliability. In our study, AIMS and PDMS-2 were administered to each infant only once, so longitudinal data for motor function, including assessments made after therapeutic intervention, were not available. Information regarding the predictive validity of AIMS was also limited. In a future study, criterion-related validity could be investigated by performing longitudinal follow-up of motor development in infants at high risk of motor delays, with the infants evaluated with AIMS and PDMS-2 at corrected ages of 6 and 12 months.

## 10. Conclusion

Our results indicate that AIMS is a reliable and stable instrument for evaluating motor function in high-risk infants in China. Because of its straightforward administration and low cost, AIMS could be used to monitor motor development during follow-up of high-risk infants. Furthermore, AIMS can guide early intervention for developmental disorders.

## Figures and Tables

**Table 1:** Characteristics of the infants participating in the study.

Parameter	Participants (N = 50)		
	n		%
Male	29		58
Female	21		42
Low birth weight:			
≥1500 g and <2500 g	14		28
<1500 g	5		10
Prematurity:			
≥32 weeks and <37 weeks	18		36
<32 weeks	7		14
Polyembryony	14		28
Asphyxia	13		26
Intracranial hemorrhage:			
grades I–II	8		16
grades III–IV	12		24
Hyperbilirubinemia	6		12
Use of ventilator	3		6
	Mean	SD	Range
Age (months)	4.14	2.02	0.17–9.27
Gestational age (weeks)	35.78	3.51	28–40
Birth weight (g)	2719.00	828.32	1190–4150
Length of hospital stay (days)	23.67	17.71	7–60

SD: standard deviation.

**Table 2:** Interrater reliability of AIMS scores recorded independently by raters A, B, and C.

Group	Rater A		Rater B		Rater C		Inter-rater reliability	
	Mean	SD	Mean	SD	Mean	SD	ICC	95%CI
0–3 months (n = 23)								
Prone	2.52	1.12	2.52	1.12	2.48	1.28	0.981	0.962–0.991
Supine	2.43	1.08	2.43	1.04	2.35	1.19	0.975	0.949–0.988
Sitting	0.78	0.80	0.65	0.78	0.70	0.77	0.948	0.895–0.976
Standing	0.78	0.42	0.78	0.42	0.87	0.46	0.946	0.891–0.975
Total score	6.52	2.76	6.39	2.73	6.39	3.07	0.982	0.963–0.992
4–9 months (n = 27)								
Prone	6.74	3.21	6.74	3.08	6.59	2.83	0.992	0.985–0.996
Supine	5.89	1.83	5.78	1.91	5.70	1.79	0.991	0.982–0.995
Sitting	4.22	3.18	4.22	3.20	4.00	3.08	0.991	0.982–0.995
Standing	1.93	1.14	1.93	1.14	1.81	1.11	0.920	0.848–0.961
Total score	18.78	8.63	18.67	8.65	18.11	7.83	0.996	0.992–0.998
0–9 months (n = 50)								
Prone	4.70	3.32	4.72	3.25	4.62	3.10	0.995	0.992–0.997
Supine	4.34	2.24	4.28	2.23	4.20	2.21	0.994	0.990–0.996
Sitting	2.60	2.96	2.56	2.99	2.46	2.84	0.992	0.988–0.995
Standing	1.42	1.03	1.42	1.03	1.40	0.97	0.943	0.909–0.966
Total score	13.06	9.02	12.98	9.02	12.68	8.45	0.997	0.995–0.998

95%CI: 95% confidence interval; AIMS: Alberta Infant Motor Scale; ICC: intraclass correlation coefficient; SD: standard deviation.

**Table 3:** Interrater reliability of AIMS scores between any two raters.

Group	Raters A and B		Raters A and C		Raters B and C	
	ICC	95%CI	ICC	95%CI	ICC	95%CI
0–3 months (n = 23)						
Prone	0.982	0.957–0.992	0.976	0.944–0.990	0.959	0.904–0.983
Supine	0.979	0.951–0.991	0.965	0.918–0.985	0.944	0.868–0.976
Sitting	0.907	0.781–0.961	0.923	0.819–0.967	0.940	0.858–0.975
Standing	1.000	1.000–1.000	0.880	0.717–0.949	0.880	0.717–0.949
Total score	0.977	0.947–0.990	0.975	0.940–0.989	0.967	0.921–0.986
4–9 months (n = 27)						
Prone	0.992	0.983–0.996	0.984	0.964–0.992	0.990	0.977–0.995
Supine	0.993	0.984–0.997	0.982	0.960–0.992	0.983	0.963–0.992
Sitting	0.990	0.979–0.996	0.987	0.972–0.994	0.979	0.955–0.991
Standing	1.000	1.000–1.000	0.814	0.592–0.915	0.814	0.592–0.915
Total score	0.999	0.997–0.999	0.991	0.981–0.996	0.992	0.982–0.996
0–9 months (n = 50)						
Prone	0.995	0.991–0.997	0.990	0.983–0.994	0.992	0.985–0.995
Supine	0.995	0.991–0.997	0.990	0.982–0.994	0.988	0.978–0.993
Sitting	0.992	0.986–0.995	0.989	0.981–0.994	0.985	0.974–0.992
Standing	1.000	1.000–1.000	0.867	0.766–0.925	0.867	0.766–0.925
Total score	0.999	0.997–0.999	0.994	0.989–0.997	0.994	0.990–0.997

95%CI: 95% confidence interval; AIMS: Alberta Infant Motor Scale; ICC: intraclass correlation coefficient; SD: standard deviation.

**Table 4:** Intrarater reliability of AIMS scores for rater B.

Group	First scoring		Second scoring		Intra-rater reliability	
	Mean	SD	Mean	SD	ICC	95%CI
0–3 months (n = 23)						
Prone	2.52	1.12	2.39	1.27	0.869	0.692–0.945
Supine	2.43	1.04	2.52	0.90	0.726	0.453–0.883
Sitting	0.65	0.78	0.65	0.83	0.673	0.430–0.861
Standing	0.78	0.42	1.04	0.56	0.580	0.319–0.822
Total score	6.39	2.73	6.61	2.79	0.872	0.699–0.946
4–9 months (n = 27)						
Prone	6.74	3.08	6.56	3.00	0.985	0.967–0.993
Supine	5.78	1.91	5.74	1.89	0.976	0.946–0.989
Sitting	4.22	3.20	4.33	2.87	0.982	0.961–0.992
Standing	1.93	1.14	2.22	0.85	0.922	0.828–0.964
Total score	18.67	8.65	18.85	8.01	0.991	0.980–0.996
0–9 months (n = 50)						
Prone	4.72	3.25	4.64	3.15	0.989	0.981–0.994
Supine	4.30	2.22	4.26	2.21	0.985	0.974–0.992
Sitting	2.92	3.93	2.64	2.85	0.988	0.979–0.993
Standing	1.42	1.03	1.68	0.94	0.923	0.864–0.956
Total score	12.98	9.02	13.22	8.69	0.995	0.991–0.990

95%CI: 95% confidence interval; AIMS: Alberta Infant Motor Scale; ICC: intraclass correlation coefficient; SD: standard deviation.

**Table 5:** Intrarater reliability of AIMS scores for rater C.

Group	First scoring		Second scoring		Intra-rater reliability	
	Mean	SD	Mean	SD	ICC	95%CI
0–3 months (n = 23)						
Prone	2.48	1.28	2.52	1.28	0.828	0.595–0.927
Supine	2.35	1.19	2.39	0.89	0.726	0.354–0.884
Sitting	0.70	0.77	0.57	0.73	0.724	0.349–0.883
Standing	0.87	0.46	1.13	0.55	0.754	0.419–0.896
Total score	6.39	3.07	6.61	2.78	0.811	0.554–0.920
4–9 months (n = 27)						
Prone	6.59	2.83	6.56	2.79	0.981	0.959–0.992
Supine	5.70	1.79	5.74	1.87	0.991	0.981–0.996
Sitting	4.00	3.08	4.30	3.04	0.977	0.950–0.990
Standing	1.81	1.11	2.19	0.88	0.728	0.403–0.876
Total score	18.11	7.83	18.78	8.06	0.992	0.983–0.997
0–9 months (n = 50)						
Prone	4.62	3.10	4.70	3.00	0.987	0.977–0.993
Supine	4.20	2.21	4.20	2.25	0.988	0.978–0.993
Sitting	2.46	2.84	2.58	2.94	0.983	0.970–0.990
Standing	1.40	0.97	1.70	0.91	0.819	0.681–0.897
Total score	12.68	8.45	13.18	8.69	0.994	0.989–0.996

95%CI: 95% confidence interval; AIMS: Alberta Infant Motor Scale; ICC: intraclass correlation coefficient; SD: standard deviation.

**Table 6:** The correlation of AIMS total score with PDMS-2 raw score for each subtest.

	RE	ST	LO	GR	VI
Value	4.40 ± 3.43	15.60 ± 8.39	13.79 ± 10.15	12.77 ± 7.98	17.53 ± 11.50
ICC	0.751	0.952	0.977	0.962	0.948
95%CI	0.553-0.861	0.914-0.973	0.959-0.987	0.931-0.979	0.907-0.971

PDMS-2 subtest scores are shown as means ± standard deviations. AIMS: Alberta Infant Motor Scale; GR: grasping; LO: locomotion; PDMS-2: Peabody Developmental Motor Scale-2; ICC: intraclass correlation coefficient of with AIMS total score; 95%CI: 95% confidence interval; RE: reflexes; ST: stationary; VI: visual-motor integration.

**Table 7:** The concordance correlations (kappa) of the fifth percentile of the Alberta Infant Motor Scale (AIMS) total score with the motor quotients of the Peabody Developmental Motor Scale-2 (PDMS-2).

	GMQ		FMQ		TMQ		N
	≥90	<90	≥90	<90	≥90	<90	
AIMS percentile							
≥5%	26	4	29	1	27	3	30
<5%	5	12	7	10	3	14	17
Kappa	0.580		0.601		0.724		

AIMS: Alberta Infant Motor Scale; FMQ: fine motor quotient; GMQ: gross motor quotient; PDMS-2: Peabody Developmental Motor Scale-2; TMQ: total motor quotient.

## Data Availability

The data used to support the findings of this study are available from the corresponding author upon request.

## Supplementary Materials

Supplementary Materials Column 1: N: case number. Column 2: gender (F: female, M: male). Column 3: age (months). Column 4: birth weight (kg). Column 5: gestational age (weeks). Column 6: A prone: the AIMS prone subsection score recorded independently by rater A. Column 7: A supine: the AIMS supine subsection score recorded independently by rater A. Column 8: A sitting: the AIMS sitting subsection score recorded independently by rater A. Column 9: A standing: the AIMS standing subsection score recorded independently by rater A. Column 10: A total score: the AIMS total score recorded independently by rater A. Column 11: A percentile: the percentile of the AIMS total score recorded independently by rater A. Column 12: B1 prone: the AIMS prone subsection score recorded independently by rater B first scoring. Column 13: B1 supine: the AIMS supine subsection score recorded independently by rater B first scoring. Column 14: B1 sitting: the AIMS sitting subsection score recorded independently by rater B first scoring. Column 15: B1 standing: the AIMS standing subsection score recorded independently by rater B first scoring. Column 16: B1 total score: the AIMS total score recorded independently by rater B first scoring. Column 17: B1 percentile: the percentile of the AIMS total score recorded independently by rater B first scoring. Column 18: C1 prone: the AIMS prone subsection score recorded independently by rater C first scoring. Column 19: C1 supine: the AIMS supine subsection score recorded independently by rater C first scoring. Column 20: C1 sitting: the AIMS sitting subsection score recorded independently by rater C first scoring. Column 21: C1 standing: the AIMS standing subsection score recorded independently by rater C first scoring. Column 22: C1 total score: the AIMS total score recorded independently by rater C first scoring. Column 23: C1 percentile: the percentile of the AIMS total score recorded independently by rater C first scoring. Column 24: ST: PDMS-2 ST subscale score. Column 25: LO: PDMS-2 LO subscale score. Column 26: RE: PDMS-2 RE subscale score. Column 27: GR: PDMS-2 GR subscale score. Column 28: VI: PDMS-2 VI subscale score. Column 29: P-GMQ: GMQ of the PDMS-2. Column 30: P-FMQ: FMQ of the PDMS-2. Column 31: P-TMQ: TMQ of the PDMS-2. Column 32: B2 prone: the AIMS prone subsection score recorded independently by rater B second scoring. Column 33: B2 supine: the AIMS supine subsection score recorded independently by rater B second scoring. Column 34: B2 sitting: the AIMS sitting subsection score recorded independently by rater B second scoring. Column 35: B2 standing: the AIMS standing subsection score recorded independently by rater B second scoring. Column 36: B2 total score: the AIMS total score recorded independently by rater B second scoring. Column 37: B2 percentile: the percentile of the AIMS total score recorded independently by rater B second scoring. Column 38: C2 prone: the AIMS prone subsection score recorded independently by rater C second scoring. Column 39: C2 supine: the AIMS supine subsection score recorded independently by rater C second scoring. Column 40: C2 sitting: the AIMS sitting subsection score recorded independently by rater C second scoring. Column 41: C2 standing: the AIMS standing subsection score recorded independently by rater C second scoring. Column 42: C2 total score: the AIMS total score recorded independently by rater C second scoring. Column 43: C2 percentile: the percentile of the AIMS total score recorded independently by rater C second scoring. AIMS: Alberta Infant Motor Scale; PDMS-2: Peabody Developmental Motor Scale-2; ST: stationary; LO: locomotion; RE: reflexes; GR: grasping; VI: visual-motor integration; GMQ: gross motor quotient; FMQ: fine motor quotient; TMQ: total motor quotient.
